# Key Metabolic Enzymes Involved in Remdesivir Activation in Human Lung Cells

**DOI:** 10.1128/AAC.00602-21

**Published:** 2021-08-17

**Authors:** Ruidong Li, Albert Liclican, Yili Xu, Jared Pitts, Congrong Niu, Jingyu Zhang, Cynthia Kim, Xiaofeng Zhao, Daniel Soohoo, Darius Babusis, Qin Yue, Bin Ma, Bernard P. Murray, Raju Subramanian, Xuping Xie, Jing Zou, John P. Bilello, Li Li, Brian E. Schultz, Roman Sakowicz, Bill J. Smith, Pei-Yong Shi, Eisuke Murakami, Joy Y. Feng

**Affiliations:** a Gilead Sciences, Inc., Foster City, California, USA; b Department of Biochemistry and Molecular Biology, University of Texas Medical Branch, Galveston, Texas, USA

**Keywords:** remdesivir, prodrug activation, CES1, CatA, HINT1, nucleotide analogs, COVID-19, SARS-CoV-2

## Abstract

Remdesivir (RDV; GS-5734, Veklury), the first FDA-approved antiviral to treat COVID-19, is a single-diastereomer monophosphoramidate prodrug of an adenosine analogue. RDV is taken up in the target cells and metabolized in multiple steps to form the active nucleoside triphosphate (TP) (GS-443902), which, in turn, acts as a potent and selective inhibitor of multiple viral RNA polymerases. In this report, we profiled the key enzymes involved in the RDV metabolic pathway with multiple parallel approaches: (i) bioinformatic analysis of nucleoside/nucleotide metabolic enzyme mRNA expression using public human tissue and lung single-cell bulk mRNA sequence (RNA-seq) data sets, (ii) protein and mRNA quantification of enzymes in human lung tissue and primary lung cells, (iii) biochemical studies on the catalytic rate of key enzymes, (iv) effects of specific enzyme inhibitors on the GS-443902 formation, and (v) the effects of these inhibitors on RDV antiviral activity against SARS-CoV-2 in cell culture. Our data collectively demonstrated that carboxylesterase 1 (CES1) and cathepsin A (CatA) are enzymes involved in hydrolyzing RDV to its alanine intermediate MetX, which is further hydrolyzed to the monophosphate form by histidine triad nucleotide-binding protein 1 (HINT1). The monophosphate is then consecutively phosphorylated to diphosphate and triphosphate by cellular phosphotransferases. Our data support the hypothesis that the unique properties of RDV prodrug not only allow lung-specific accumulation critical for the treatment of respiratory viral infection such as COVID-19 but also enable efficient intracellular metabolism of RDV and its MetX to monophosphate and successive phosphorylation to form the active TP in disease-relevant cells.

## INTRODUCTION

**FIG 1:**
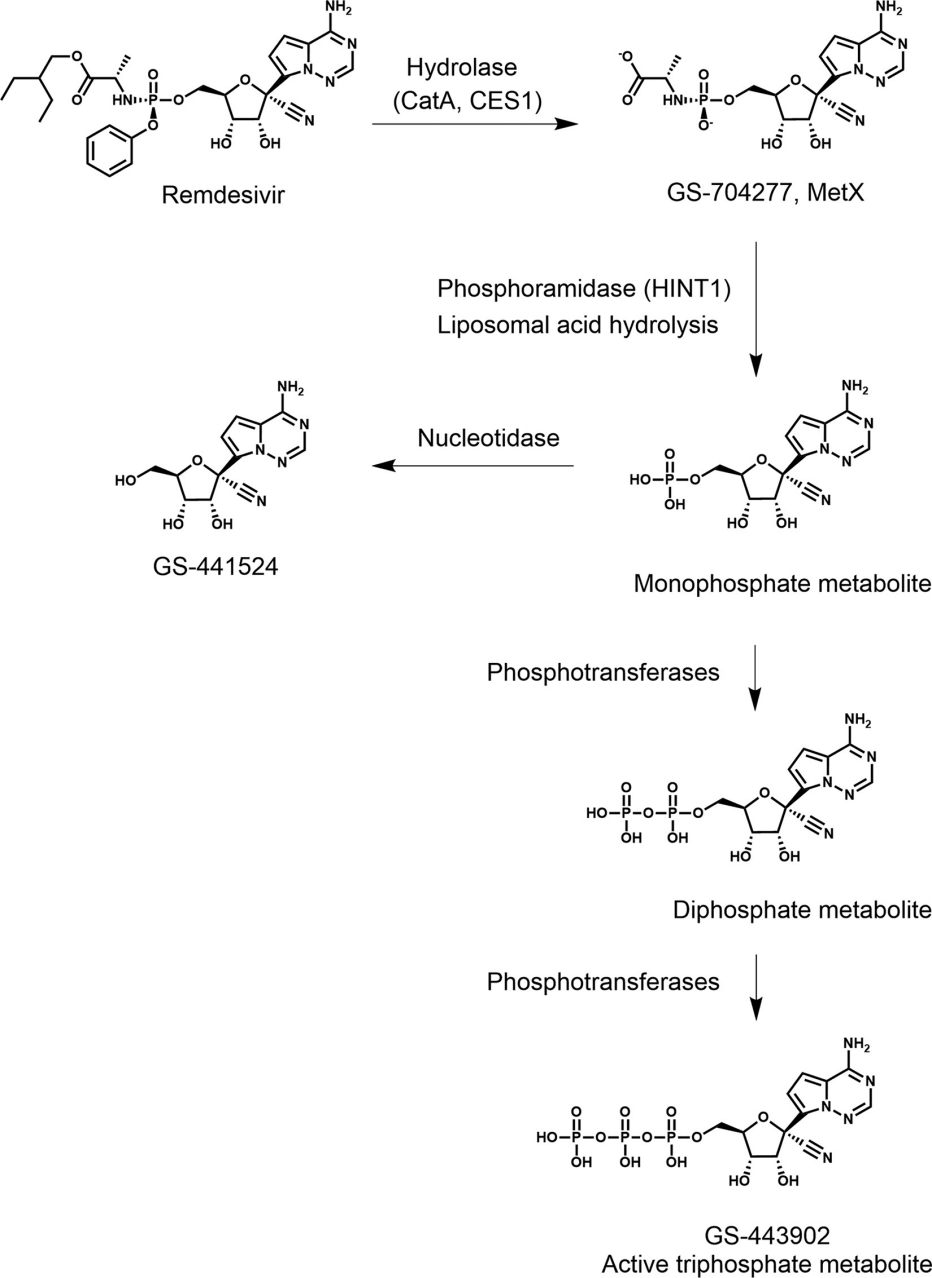
Structures of RDV and major metabolites.

RDV is a single-diastereomer monophosphoramidate prodrug of an adenosine analog (Fig. 1). It has broad-spectrum activity against coronaviruses (SARS-CoV-2, SARS-CoV, and MERS-CoV) (1, 2) and is the first FDA-approved antiviral for the treatment of coronavirus disease 2019 (COVID-19) (Veklury). RDV is intracellularly metabolized to its active triphosphate form (GS-443902; Fig. 1) by cellular metabolic enzymes and kinases. Additional intracellular metabolites include an alanine metabolite (MetX), parent nucleoside (GS-441524), and mono-and diphosphate nucleotides (Fig. 1). Efficient activation of RDV in the lung is critical for treating respiratory viral infections such as COVID-19. Previous studies by Birkus and Murakami have elucidated key enzymatic reactions and processes in phosphoramidate prodrug activation pathways (3–9). Based on these findings, we focused our studies on the enzymes that facilitate these critical reactions, namely, carboxylesterase 1 (CES1), cathepsin A (CatA), and histidine triad nucleotide-binding protein 1 (HINT1); gene expression of 11 other potential enzymes; as well as the process of lysosomal acid hydrolysis. After removal of the RDV promoiety, the resulting nucleoside monophosphate (NMP) metabolite is phosphorylated to its nucleoside diphosphate (NDP) and, ultimately, to the active nucleoside triphosphate (NTP) by multiple host phosphotransferases (10).

In this study, we investigated the contributions of the aforementioned enzymes and metabolic processes using the following multiple parallel approaches: (i) mRNA expression in human lung tissue and single cells from public databases, (ii) mRNA and protein expression in primary human lung cells and human lung postmitochondrial supernatant (S9) fractions, (iii) rates of catalysis by recombinant proteins, (iv) the effects of metabolic enzyme inhibitors on RDV metabolism in primary human lung cells, and (v) the effects of these inhibitors on RDV SARS-CoV-2 potency in cell culture.

## RESULTS

### Pulmonary mRNA expression of enzymes involved in activation of nucleoside and nucleotide prodrugs.

### (i) Pulmonary expression of enzymes involved in the metabolism of phosphoramidate prodrugs.

**FIG 2:**
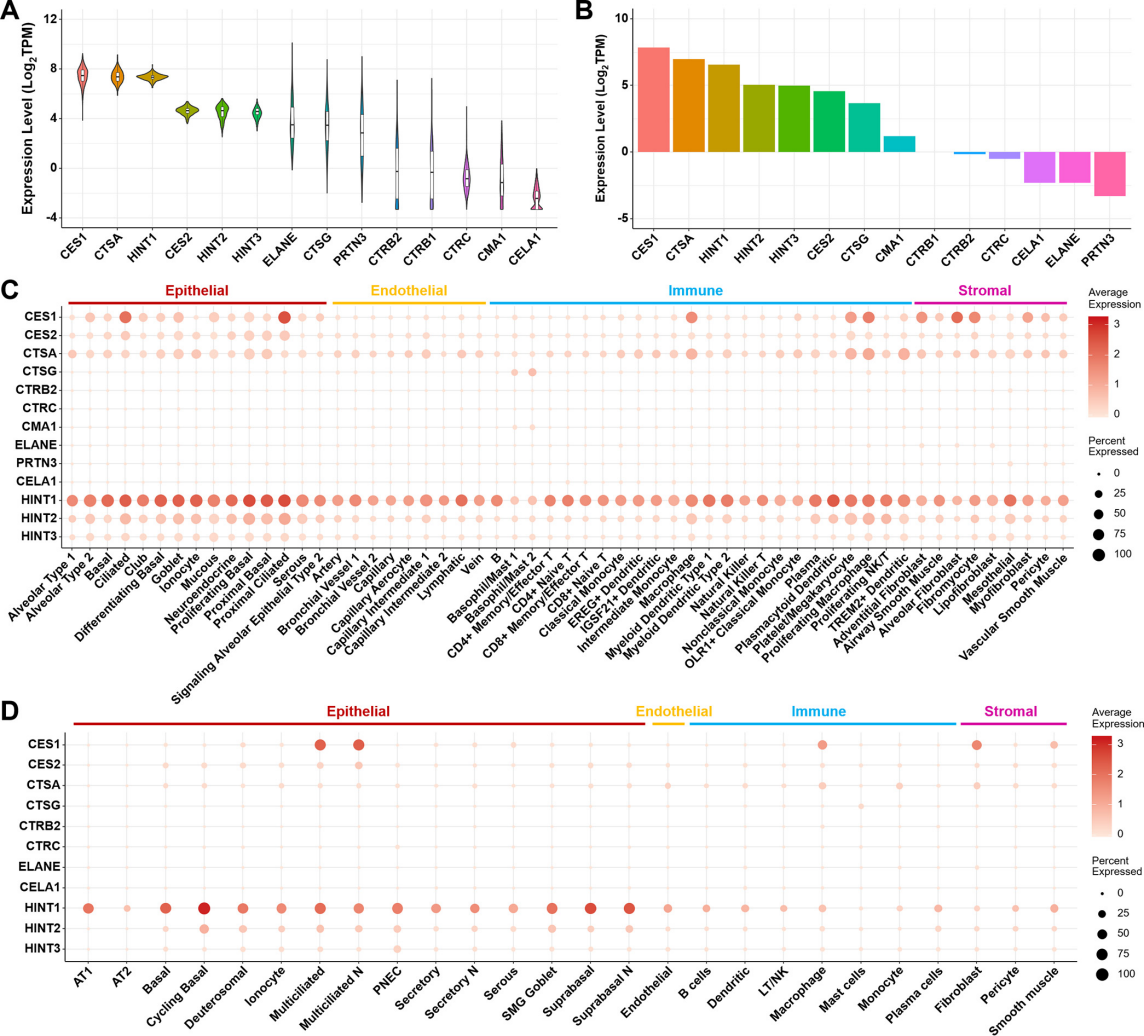
Expression of enzymes involved in the metabolism of phosphoramidate prodrug in human lung tissue and in single cells of human lung and airways. (A) Bulk mRNA expression of genes in normal human lung tissue in the GTEx data set. (B) Bulk mRNA expression of genes in normal human lung tissue in the HPA data set. (C) Expression profiles of genes in human healthy lung cells based on scRNA-seq data. (D) Expression profiles of genes in human healthy airway cells based on scRNA-seq data.

We comprehensively investigated the expression of 14 genes involved in phosphoramidate prodrug metabolism using both bulk mRNA sequence (RNA-seq) data from normal human lung tissue and single-cell RNA-seq data from normal human lung and airways. The genes of interest were *CES1*, *CES2*, *CTSA*, *CTSG*, *CTRB1*, *CTRB2*, *CTRC*, *CMA1*, *ELANE*, *PRTN3*, *CELA1*, *HINT1*, *HINT2*, and *HINT3* (Table 1). Data for this analysis were pulled from two comprehensive public bulk RNA-seq data resources for nondiseased human tissues, the Genotype-Tissue Expression (GTEx) and the Human Protein Atlas (HPA) (11, 12). In total, 578 and 9 normal human lung tissue samples were analyzed from the GTEx and HPA databases, respectively. Transcription per million (TPM) was used to differentiate levels of expression: TPM of <1 is considered low expression, TPM between 1 and 100 is considered medium expression, and TPM of >100 is considered high expression. Of the 14 genes evaluated, *CES1*, *CTSA* (the gene encoding CatA), and *HINT1* are highly expressed in human lung samples in both the GTEx and HPA data sets (Fig. 2A and B). Abundant levels of gene expression in human lung cells were also observed for *CES2*, *HINT2*, and *HINT3* in both data sets. The expression patterns of the aforementioned genes were consistent across the two data sets. Other genes were only detected with relatively low expression levels and large variations.

**TABLE 1:** Genes involved in activation of nucleoside monophosphate phosphoramidate prodrugs to triphosphate active metabolites

Catalytic step	Enzyme family	Enzyme(s)	Gene(s)
Prodrug→MetX	Carboxylesterase	CES1, CES2	*CES1, CES2*
		CatA	*CTSA*
	Serine protease	CatG	*CTSG*
		Chymotrypsin (A, B1, B2, C)	*CTR* (*CTRA*, *CTRB1*, *CTRB2*, *CTRC*)
		Chymase/master cell protease 1	*CMA1*
		Leukocyte elastase/neutrophil elastase/ELANE	*ELANE*
		Proteinase 3	*PRTN3*
		Pancreatic elastase I/chymotrypsin-like elastase family, member 1	*CELA1*
MetX→NMP	Phosphoramidase	HINT1, HINT2, HINT3	*HINT1*, *HINT2*, *HINT3*
Nucleoside→NMP	Phosphotransferase	Adenosine kinase	*ADK*
		High Km 5′-nucleotidase/ectonucleotidase CD73	*NT5E*
		Deoxyguanosine kinase	*DGUOK*
		Deoxycytidine kinase	*DCK*
		Thymidine kinase (TK1, TK2)	*TK1*, *TK2*
NMP→NDP	Phosphotransferase	Adenylate kinase (AK1–AK9)	*AK1*–*AK9*
		Guanylate kinase/deoxyguanylate kinase/GMP kinase	*GUK1*
		NMP kinase/nucleoside-phosphate kinase	*NDK*
NDP→NTP	Phosphotransferase	NDP kinase/NDPK/nucleoside-diphosphate kinase	*NME1*–*NME7*
		3-phosphoglycerate kinase/3-PG kinase	*PGK1*
		Creatine kinase/CKB in brain/CKM in muscle	*CKB*, *CKM*
		Pyruvate kinase	*PKM*

To evaluate the gene expression profiles at a cell type-specific level, we investigated the expression patterns of the 14 genes in two published single-cell RNA-seq data sets for normal human lung and airways (13, 14). In the molecular cell atlas of the human lung, 7 samples were collected from histologically normal lung tissue from bronchi (proximal), bronchiole (medial), and alveolar (distal) regions in 3 patients. A total of 65,662 cells were captured and sequenced based on the 10× Genomics droplet technology, and 58 transcriptionally distinct cell populations were identified. Separately, the single-cell atlas of healthy human airways was collected from 35 distinct locations from the nose to the 12th division of the airway tree in 10 healthy living donors. A total of 77,969 individual cells were captured for the human airway analysis and sequenced using the 10× Genomics droplet technology. The results of single-cell RNA-seq data analysis were largely consistent with the bulk RNA-seq data sets from HPA and GTEx. Among the 14 genes, *CES1*, *CTSA*, and *HINT1* were highly expressed in many cell types, while *CES2*, *HINT2*, and *HINT3* were only detected in a few subsets of human lung cells (Fig. 2C). Similar to the bulk analysis, the remaining 8 genes showed low levels of expression in human lung cells. A closer look into the expression of the three highly expressed genes, *CES1*, *CTSA*, and *HINT1*, in human lung cells revealed distinct expression patterns across cell types. *CES1* was expressed in all types of epithelial cells, especially ciliated cells, but not expressed in endothelial cells. *CES1* was also highly expressed in some immune cell populations (i.e., macrophages) and stromal cells (i.e., fibroblasts). *CTSA* and *HINT1* were expressed across all human lung cell types, but the expression of *HINT1* was considerably more prominent than *CTSA* in most cell types. The expression patterns of the genes were similar in human airways to that in human lung cells (Fig. 2D).

### (ii) Expression of enzymes involved in metabolism of nucleoside, NMP, NDP, and NTP in human lung tissue and lung cells.

Using the same approach described above, we also comprehensively investigated the expression of genes involved in the activation of nucleoside to the NMP, NDP, and NTP metabolites based on the GTEx, HPA, and the two single-cell RNA-seq data sets of human lung and airways.

**FIG 3:**
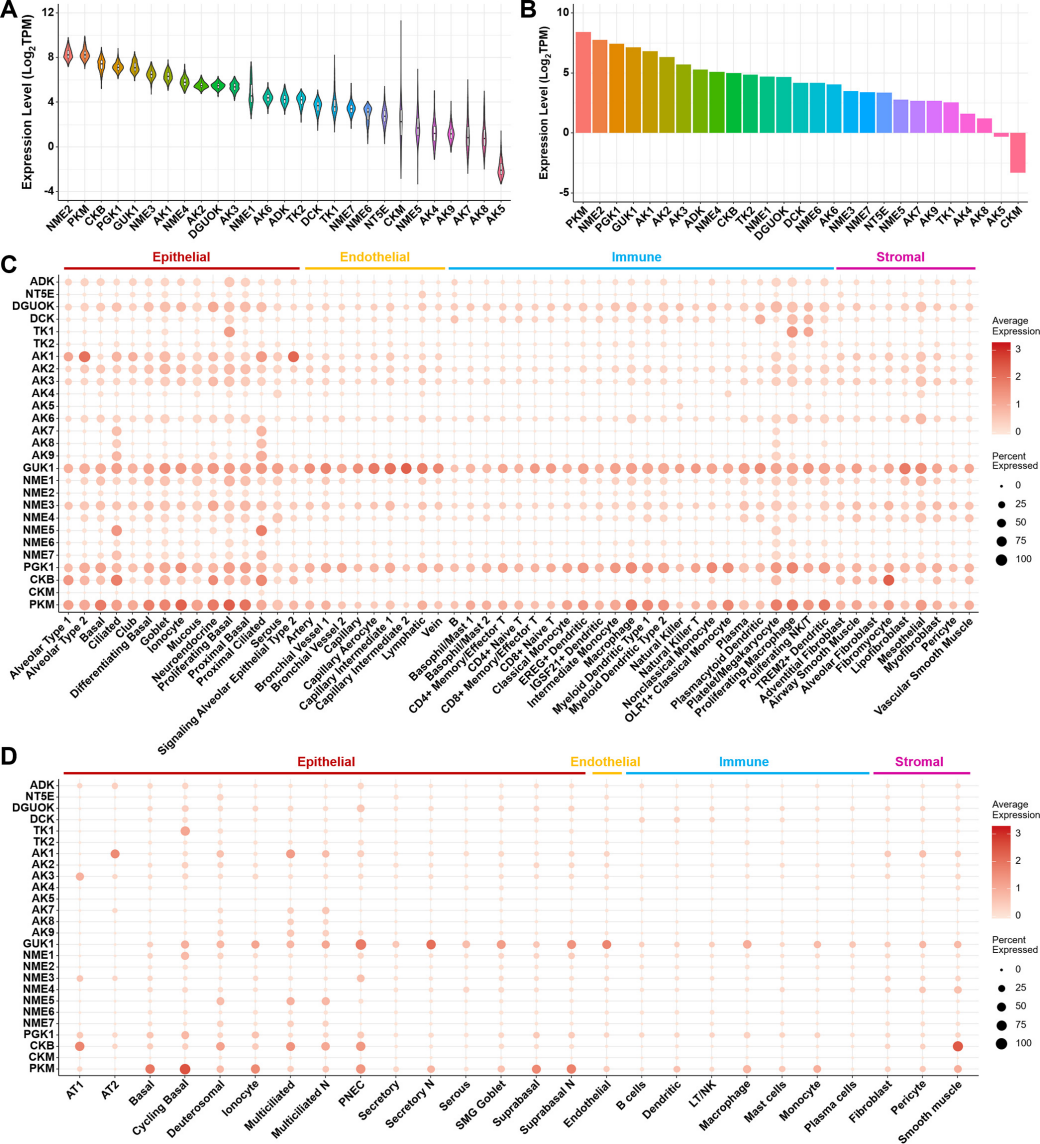
Expression of enzymes involved in metabolism of nucleoside, NMP, NDP, and NTP in human lung tissue and in single cells of human lung and airways. (A) Bulk mRNA expression of genes in normal human lung tissue in the GTEx data set. (B) Bulk mRNA expression of genes in normal human lung tissue in the HPA data set. (C) Expression profiles of genes in human healthy lung cells based on scRNA-seq data. (D) Expression profiles of genes in human healthy airway cells based on scRNA-seq data.

A total of 27 genes, including *ADK*, *NT5E*, *DGUOK*, *DCK*, *TK1*, *TK2*, *AK1-AK9*, *GUK1*, *NME1-NME7*, *PGK1*, *CKB*, *CKM*, and *PKM*, were analyzed (Table 1). Most of the genes were expressed in human lung tissue based on the bulk RNA-seq data sets (Fig. 3A and B). *NME2*, *PKM*, *CKB*, *PGK1*, and *GUK1* were the top 5 highly expressed genes in the GTEx data set, and four of them (*NME2*, *PKM*, *PGK1*, and *GUK1*) were also in the list of top 5 highly expressed genes in the HPA data set. Comparing the two enzymes ADK and NT5E, which can convert adenosine to AMP, the expression level of *ADK* was relatively higher than *NT5E*. For the nine adenylate kinase isozymes AK1 to AK9, which can convert AMP to ADP, four of them (i.e., *AK1*, *AK2*, *AK3*, and *AK6*) had relatively higher expression levels than the other five genes (i.e., *AK4*, *AK5*, *AK7*, *AK8*, and *AK9*). The expression level of *NME2* was the highest among the seven nucleoside diphosphate kinase isozymes, whereas the other isozymes were also moderately expressed in human lung tissue. Generally, the results of single-cell RNA-seq data analysis were also consistent with the bulk RNA-seq data sets, except that *NME2* was not highly expressed in human lung or airway cells in the single-cell RNA-seq data sets (Fig. 3C). The other highly expressed genes, *PKM*, *PGK1*, and *GUK1*, were broadly expressed in almost all of the human lung cell populations. *CKB* was highly expressed in many subsets of epithelial cells, but not endothelial cells. The expression of *ADK* in human lung epithelial cells was higher than *NT5E*. The adenylate kinase isozymes, *AK1*, *AK2*, and *AK3*, were highly expressed in many subsets of epithelial cells and also expressed in some endothelial cells, whereas *AK7*, *AK8*, and *AK9* were only specifically expressed in ciliated epithelial cells. For the seven nucleoside-diphosphate kinase isozymes, as has been demonstrated, the expression level of NME2 was low in the single-cell RNA-seq data sets. *NME1*, *NME3*, and *NME4* were broadly expressed in many subsets of epithelial and endothelial cells, whereas the expression of *NME5* and *NME7* was specific to ciliated epithelial cells. In general, the expression patterns of these genes were similar in human airways compared with human lung cells (Fig. 3D).

### CES1, CatA, and HINT1 mRNA levels in normal human bronchial epithelial cells.

**FIG 4:**
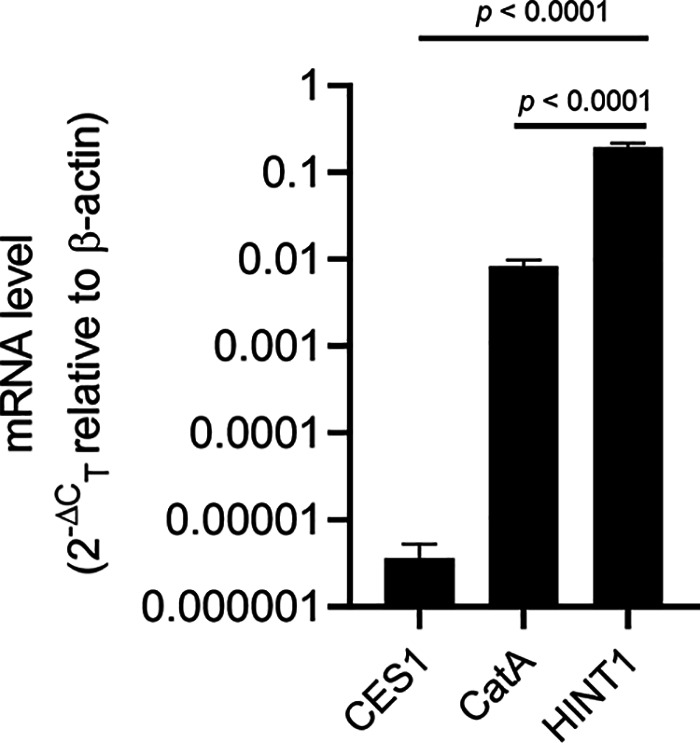
mRNA expression of CES1, CatA, and HINT1 in human NHBE cells. The levels are shown as average ± standard deviation from three independent measurements. One-way ANOVA was used for statistical analysis.

Cellular mRNA levels of CES1, CatA, and HINT1 were quantified using one-step reverse transcriptase quantitative PCR (RT-qPCR) and normalized with the mRNA level of the housekeeping gene β-actin. As summarized in Table 2 and Fig. 4, mRNA levels of CES1, CatA, and HINT1 were consistent among three different donors, with HINT1 showing the highest mRNA expression level among the three proteins (*P < *0.0001).

**TABLE 2:** Protein and mRNA expression of key enzymes in NHBE and human lung and liver S9 factions

Sample	Protein level (ng/μg total protein)^*a*^ of:			mRNA level (2^−ΔΔ^*^CT^* relative to β-actin)^*a*^ of:		
	CES1	CatA	HINT1	*CES1*	*CTSA*	*HINT1*
NHBE	<0.1	0.33 ± 0.10	1.0 ± 0.3	3.7 ± 1.6 × 10^−6^	8.3 ± 1.6 × 10^−3^	0.19 ± 0.02
Human lung S9	2.1 ± 0.9	0.220 ± 0.004	2.4 ± 1.2	NA^*b*^	NA	NA
Human liver S9	34 ± 1	0.23 ± 0.11	4.5 ± 1.7	NA	NA	NA

a Values represent average ± SD from three donors.

b NA, not applicable.

### CES1, CatA, and HINT1 protein expression in normal human bronchial epithelial cells and human lung S9 and liver S9 fractions.

**FIG 5:**
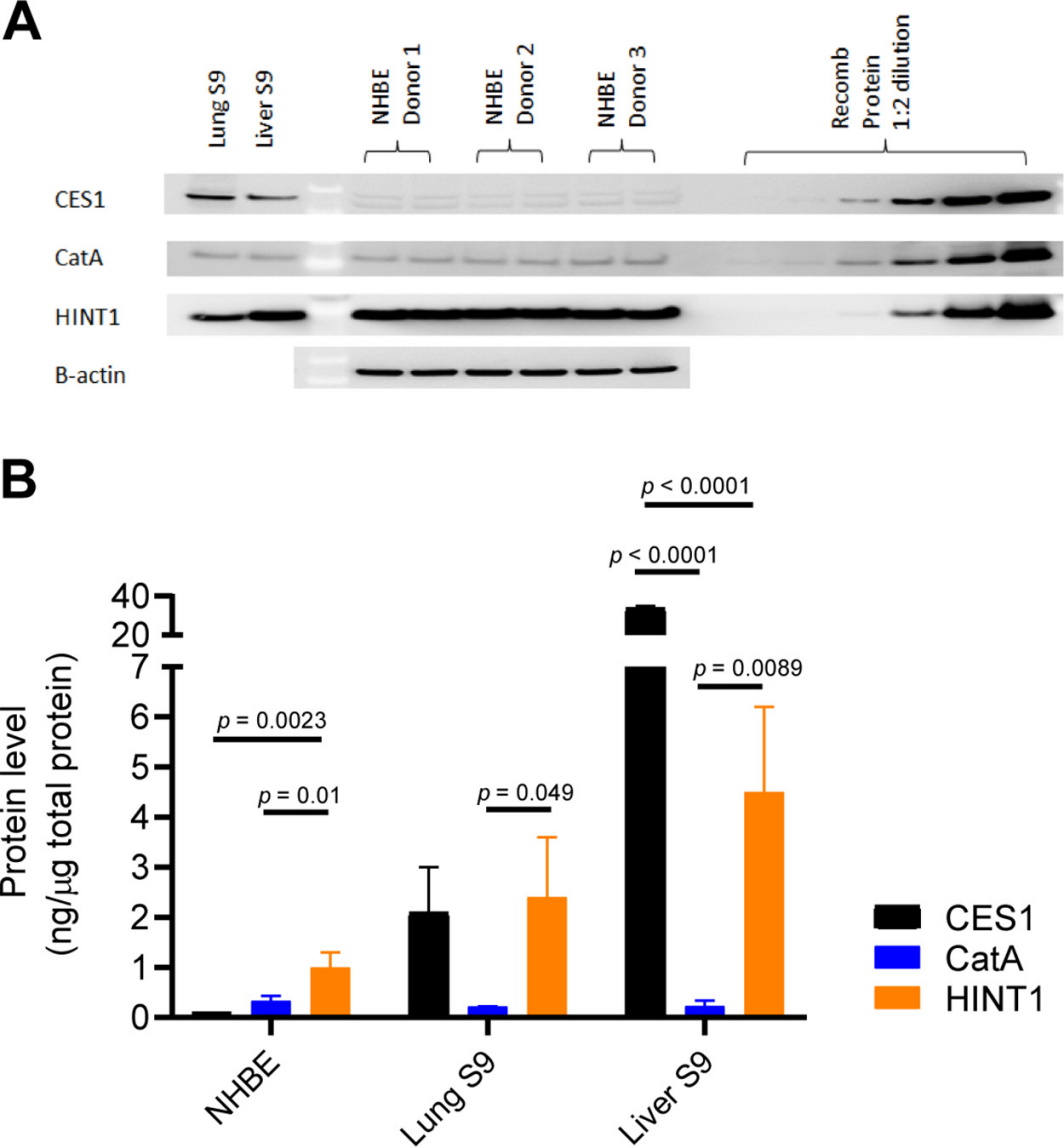
Protein expression of CES1, CatA, and HINT1 in lung NHBE cells and human lung and liver S9 fractions. (A) Western blot analysis of human cell and tissue samples. The amounts of lung S9 samples loaded for detection of CES1, CatA, and HINT1 proteins were 5 μg, 10 μg, and 10 μg, respectively. The amounts of human liver S9 samples loaded for detection of CES1, CatA, and HINT1 proteins were 0.2 μg, 10 μg, and 10 μg, respectively. The recombinant proteins were loaded at a range of 0.3 to 100 ng per lane. (B) Graphical representation of the protein levels. The levels are shown as average ± standard deviation from three independent measurements. One-way ANOVA was used for statistical analysis.

To determine if the relative mRNA levels were translated into protein expression, CES1, CatA, and HINT1 proteins were extracted from normal human bronchial epithelial (NHBE) cells, human lung S9 fraction, and liver S9 fraction and quantified using Western blot analysis with standard curves generated from recombinant human proteins. As summarized in Table 2 and Fig. 5, the levels of CES1, CatA, and HINT1 proteins in NHBE cells were highly consistent among the three different donors, with HINT1 as the highest expressed among the three proteins (*P* values range from 0.0023 to 0.01), consistent with the mRNA expression levels observed in these cells. In the human lung S9 fraction, both CES1 and HINT1 were highly expressed. In contrast, in the human liver S9 fraction, CES1 protein was the most highly expressed, and the rank order of protein expression was CES1 was greater than HINT1, which was greater than CatA (*P* values from <0.0001 to 0.0089), consistent with the reported high expression level of liver CES1 (15).

### Biochemical evaluation of ester-bond cleavage by CES1, CES2, and CatA.

The catalytic activity of carboxylesterase 1b (CES1b), carboxylesterase 1c (CES1c), CES2, and CatA toward RDV hydrolysis was measured using protein-enriched subcellular fraction (CES isozymes) or recombinant proteins (CatA). The reaction rates were determined by monitoring the disappearance of RDV using a liquid chromatography-mass spectrometry (LC-MS) method. The results are summarized in Table 3. For the CES-catalyzed RDV hydrolysis, both CES1b and CES1c hydrolyzed RDV with rates of (4.8 ± 0.4) × 10^−2^ and (6.1 ± 0.4) × 10^−2 ^nmol/min/mg. These rates were 2 and 4 times faster than the positive-control compound oseltamivir, respectively. In contrast, RDV was a poor substrate for CES2, with a half-life longer than 16 h. The rate of CatA-catalyzed RDV hydrolysis was determined to be 74 ± 32 nmol/min/mg, 3.4-fold lower than the hydrolysis rate of tenofovir alafenamide fumarate (TAF). In this study, TAF and oseltamivir, known substrates of CES1, CES2, or CatA, were hydrolyzed at rates similar to those reported in prior publications after the rates were normalized to 1 μM substrate concentration (6, 16).

**TABLE 3:** Hydrolysis of RDV by key metabolic enzymes^*a*^

Compound	Activity for amidate ester cleavage (nmol/min/mg) (*t*_1/2_ [min]) for enzyme:			
	CES1b	CES1c	CES2	CatA
RDV	4.8 ± 0.4 × 10^−2^ (9.7 ± 0.8)	6.1 ± 0.4 × 10^−2^ (7.7 ± 0.5)	2.8 ± 2.6 × 10^−4^ (> 1000)	74 ± 32 (43 ± 25)
Control				
Oseltamivir	2.3 ± 0.1 × 10^−2^ (20 ± 1)	1.5 ± 0.2 × 10^−2^ (31 ± 4)		
Procaine			4.4 ± 0.8 × 10^−3^ (110 ± 20)	
TAF (GS-7340)				254 ± 154 (13 ± 6)

a Values represent the average ± standard deviation from three independent measurements. *t*_1/2_, half-life.

### Biochemical evaluation of phosphoramide (P-N) bond cleavage by HINT1-catalyzed and lysosomal-mimicking acid hydrolysis.

The HINT1-catalyzed hydrolysis of the RDV intermediate metabolite, MetX, was measured using recombinant protein, and the reaction rates were determined by monitoring the disappearance of RDV MetX using LC-MS. The results are summarized in Table 4. Compared to a known substrate of HINT1 (GS-6620 MetX), the RDV MetX was metabolized 6 times faster, suggesting that HINT1 is a key enzyme in RDV activation (8). P-N bond cleavage of RDV MetX was evaluated under acidic pH, mimicking intracellular lysosomal conditions. As summarized in Table 4, no RDV MetX hydrolysis product was detectable, suggesting that chemical hydrolysis is minimally involved in the RDV activation pathway.

**TABLE 4:** Hydrolysis of RDV MetX and control compounds by HINT1 or acidic hydrolysis^*a*^

Compound	HINT1 hydrolysis (nmol/min/mg) (*t*_1/2_ [min])	Lysosomal hydrolysis at pH 4.5 (nmol/min) (*t*_1/2_ [min])
RDV MetX	32 ± 12 (5.5 ± 2.3)	0 ± 0 (>10^4^)
Control		
GS-6620 MetX	5.3 ± 2.4 (38 ± 30)	
TAF MetX		1.2 ± 0.6 × 10^−3^ (16 ± 7)

a Values represent the average ± standard deviation of three independent measurements.

### Biochemical evaluation of small-molecule inhibitors of CES1, CES2, CatA, and HINT1 enzymes.

We evaluated the specificity of four small-molecule inhibitors against CES1, CES2, CatA, and HINT1 in biochemical assays. As summarized in Table 5, bis(4-nitrophenyl)phosphate (BNPP) and telaprevir were specific inhibitors of CES and CatA, respectively, and their observed concentration causing 50% decrease in product formation in the biochemical assays (IC_50_) values (BNPP IC_50_ values range from 0.7 to 14 μM for CES1b, CES1c, and CES2, and telaprevir IC_50_ values range from 0.078 to 0.34 μM for CatA) were consistent with published data (BNPP IC_50_, 0.11 μM for CES1 and telaprevir IC_50_, 0.21 μM for CatA) (7, 9). The two reported HINT1 inhibitors, TrpGc and the nucleoside acyl-sulfamate compound 7, inhibited HINT1, with IC_50_ values of 1.5 μM and 0.16 μM, respectively, consistent with data previously reported by Shah et al. (TrpGc dissociation constant [*K_d_*], 3.65 μM; compound 7 *K_d_*, 0.23 μM) (17).

**TABLE 5:** Evaluation of enzyme inhibitors in biochemical assays

Inhibitors	Inhibition (IC_50_) of enzyme activities (μM)^*a*^^,^^*b*^ of:					
	CES1b	CES1c	CES2	CatA^*c*^		HINT1
				pH 6.5	pH 5.5	
BNPP	0.75 ± 0.12	14.0 ± 5.6	1.2 ± 0.2	>100	>100	>100
Telaprevir	>100	>100	>100	0.078 ± 0.009	0.34 ± 0.06	>100
TrpGc	>100	>100	>100	>100	>100	1.5 ± 0.5
Compound 7	>100	>100	>100	>100	>100	0.16 ± 0.05

a Values represent the average ± standard deviation of three independent measurements.

b Substrates for CES isozymes, CatA, and HINT1 were 4-NPA (4-nitrophenyl acetate), fluorogenic peptide [MCA-Arg-Pro-Pro-Gly Phe-Ser-Ala-Phe-Lys(DNP)-OH], and RDV MetX, respectively.

c CatA assay was conducted under both pH 6.5 and pH 5.5. The pH 6.5 condition was used to minimize potential acid hydrolysis of RDV. The pH 5.5 condition was used to resemble the native acidic lysosomal environment for this enzyme.

### Effects of hydrolase inhibitors on RDV activation in NHBE.

**FIG 6:**
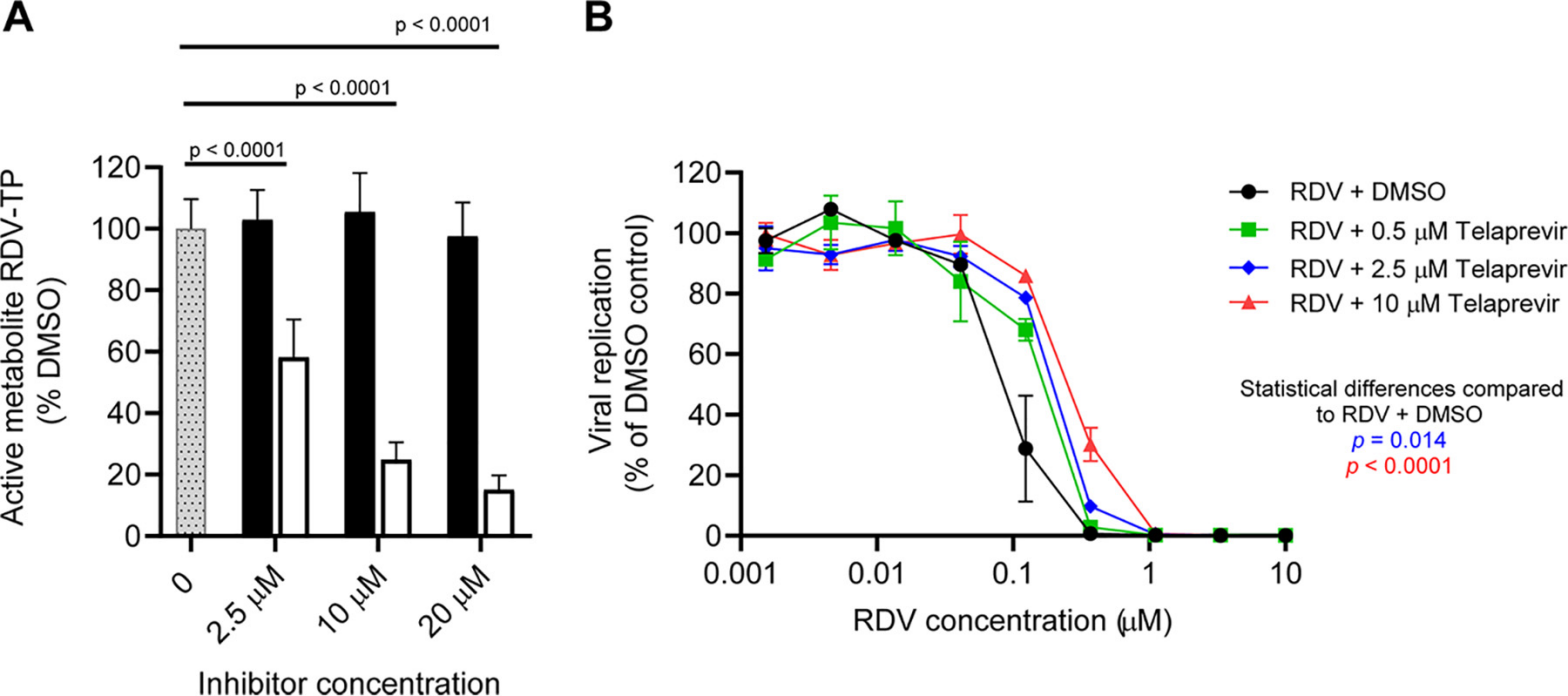
Effect of CES1 inhibitor BNPP and CatA inhibitor telaprevir on formation of active metabolite GS-443902 in RDV-treated NHBE (A) and antiviral activity against SARS-CoV-2 in A549-hACE2 cells (B). (A) Cells from three different donors were incubated with 1 μM RDV and DMSO (gray dotted bar), BNPP (filled bar), and telaprevir (open bar) and harvested at 24 h post-compound addition. Triphosphate (TP) levels are shown as average ± standard deviation of the percentage of the DMSO control across the three donors. One-way ANOVA was used for statistical analysis. (B) Effect of increasing telaprevir concentrations on potency of RDV against SARS-CoV-2 replication in A549-hACE2 cells. The RDV titration curve in the presence of either DMSO alone (black circles) or 0.5 (green boxes), 2.5 (blue diamonds), or 10 μM telaprevir (red triangles) from one of three experiments is displayed. The levels are shown as average ± standard deviation from three replicates. Two-way ANOVA was used for statistical analysis.

To assess the impact of CES1, CatA, and HINT1 activity in the metabolic activation of RDV to its active triphosphate in NHBE cells from multiple donors, GS-443902 formation was assessed following coincubation of RDV with the specific inhibitors of these enzymes across multiple concentrations (Fig. 6A and Fig. S1 in the supplemental material). Cells were harvested at 24 h after compound addition, and the GS-443902 levels were assessed by an LC-MS method. While BNPP did not cause a significant change in GS-443902 formation at the highest inhibitor concentrations tested, telaprevir caused a dose-dependent inhibition of GS-443902 formation in NHBE cells *in vitro* (*P < *0.0001), indicating that CatA is the primary enzyme responsible for activation of RDV in NHBE cells. In this study, neither TrpGc nor compound 7, two HINT1 inhibitors in the biochemical assays, showed any effect on the cellular activation of sofosbuvir (SOF), a known HINT1 substrate (data not shown).

### Effects of a CatA inhibitor on RDV anti-SARS-CoV-2 activity in A549-hACE cells.

In this study, we evaluated the consequence of inhibiting CatA-mediated formation of GS-443902 on the SARS-CoV-2 antiviral activity of RDV in A549-human angiotensin-converting enzyme 2 (hACE2) human epithelial lung cells. The antiviral potency of RDV and telaprevir treatment alone against SARS-CoV-2 was approximately 99 nM and >10,000 nM, respectively (Table 6). The low antiviral activity of telaprevir is consistent with published data (18). In the presence of telaprevir, a CatA inhibitor, RDV lost its antiviral potency in a dose-dependent manner, as demonstrated by rightward shifts in the virus replication titration curves (Fig. 6B). Higher compound concentration for reducing 50% of luciferase signal (EC_50_) values than RDV-alone treatment was observed with increasing concentrations of telaprevir (*P = *0.014 at 2 μM and *P < *0.0001 at 10 μM). At the highest concentration of 10 μM telaprevir, RDV showed a 2.9-fold increase in the EC_50_ value relative to the dimethyl sulfoxide (DMSO) control (Table 6). Cytotoxicity was not observed with RDV alone or in combination with telaprevir up to 10 μM (data not shown).

**TABLE 6:** RDV antiviral activity against SARS-CoV-2 in A549-hACE2 cells in the presence of a CatA inhibitor

Parameter	Antiviral activity of:				
	RDV^*a*^	Telaprevir	RDV + 0.5 μM telaprevir	RDV + 2.5 μM telaprevir	RDV + 10 μM telaprevir
EC_50_ (nM)^*b*^	99 ± 17	>50,000	166 ± 8	223 ± 24	285 ± 29
Fold-RDV EC_50_^*b*^^,^^*c*^			1.70 ± 0.19	2.27 ± 0.25	2.91 ± 0.65

a Cultures were treated with RDV and DMSO to the same final concentration of DMSO as cultures treated with telaprevir. Cellular toxicity was monitored in parallel using CellTiter-Glo (Promega). Minimal loss of cell viability was detected at the highest concentrations of compounds tested, with the exception of telaprevir, where a CC_50_ of 29.2 μM was observed.

b Values represent average ± standard deviation of three independent measurements.

c Fold-RDV EC_50_ was calculated by dividing the EC_50_ of the telaprevir combination treatment by the EC_50_ of RDV (plus DMSO). Presented are the average fold-RDV EC_50_ of three individual experiments.

## DISCUSSION

Nucleoside analogs have played a vital role in the treatment of viral infections caused by DNA viruses, such as herpesviruses and hepatitis B virus (HBV), as well as the RNA viruses human immunodeficiency virus (HIV), hepatitis C virus (HCV), influenza virus, and SARS-CoV-2 (19–21). The nucleoside analogs are intracellularly metabolized to active 5′-triphosphate forms, which then act as potent inhibitors of viral DNA or RNA polymerases. The formation of sufficient 5′-triphosphate in relevant tissues where viral replication occurs has been made possible by the discovery of nucleotide monophosphate prodrugs (22, 23). In some cases, antiviral nucleotide monophosphate prodrug approaches have proven successful in the clinic, including the phosphodiester prodrug tenofovir disoproxil fumarate (TDF for HIV-1 and HBV), the phosphonoamidate prodrug tenofovir alafenamide fumarate (TAF for HIV-1 and HBV), as well as the phosphoramidate prodrugs sofosbuvir (SOF for HCV) and remdesivir (RDV for SARS-CoV-2).

To investigate the pulmonary expression profiles of genes involved in the metabolism of RDV, its parent nucleoside, and the nucleotide metabolites (NMP, NDP, and NTP), we comprehensively analyzed publicly available bulk RNA-seq data sets for normal human lung tissue and small cytoplasmic RNA (scRNA)-seq data sets for normal human lung and airway cells. Our analysis showed that *CES1*, *CTSA*, and *HINT1* were highly expressed in human lung tissue based on the GTEx and HPA data sets—two of the largest published bulk RNA-seq data resources for nondiseased human tissues. The expression patterns of the genes of interest were mostly consistent across the two data sets. Furthermore, we extended the analysis to a single-cell level using two high-quality single-cell RNA-seq data sets, (i) the molecular cell atlas of the human lung, containing transcriptomes of 65,662 cells from 7 histologically normal lung tissue samples; and (ii) the single-cell atlas of the human healthy airways, containing 77,969 cells at 35 distinct locations. Overall, our analysis from these two scRNA-seq data sets was largely consistent with that from the two bulk RNA-seq data sets (HPA and GTEx), further strengthening our conclusion that genes coding for RDV-metabolizing enzymes CES1, CatA, and HINT1 are highly expressed in lung cells permissive to SARS-CoV-2 infection and replication (24, 25).

RDV, SOF, and TAF, being structurally and functionally distinct, are all activated by different target tissues, and the enzymes involved in the activation pathway may also vary. In peripheral blood mononuclear cells (PBMCs), the infection and replication sites for HIV, the first ester hydrolysis of TAF is primarily catalyzed by the lysosomal enzyme CatA (26). The resulting intermediate metabolite is then chemically converted to tenofovir monophosphate (TFV-MP) under acidic lysosomal conditions (5). In treating viral infections in the liver, TAF (for HBV) and SOF (for HCV) are also efficiently activated in hepatocytes, where the ester bonds are primarily cleaved by CES1 with minor contribution from CatA (7, 9). Differences in enzyme expression in various cell types can explain the different pathways of prodrug hydrolysis. For example, while CatA is ubiquitously expressed, high levels of CES1 predominantly expressed in the liver contribute to efficient hydrolysis of TAF and SOF in hepatocytes (27, 28). In this study, the *CES1* protein expression in lung S9 fraction and the gene expression analysis of bulk- and single-cell databases are highly consistent with the published data on high levels of *CES1* expression in lung tissue (29). Therefore, it is likely that RDV is cleaved by both CES1 and CatA in human lung.

Since NHBEs are primary lung cells, we expected the enzyme expression patterns to be similar to those observed in the lung tissues. However, our analyses of mRNA and protein expression indicated that CES1 is poorly expressed in NHBE cells. Additionally, our cellular metabolism and antiviral data using specific inhibitors of CES1 and CatA clearly demonstrated that RDV is primarily hydrolyzed by CatA in NHBE cells. Consistent with our finding, Oesch et al. reported that *CES1* expression levels varied across different lung cell models *in vitro*, with little to no expression in human bronchial epithelial cells, yet it was highly expressed in lung tissues (30). The disconnect in enzyme expression between lung tissue and NHBE cells may result from differences of expression of the enzymes in different parts of the lung or suppression of *CES1* expression during the isolation and/or by the cell culture conditions of NHBE cells. Therefore, in lung tissue *in vivo*, it is likely that the RDV ester is cleaved by both CES1 and CatA. One must be careful when evaluating the antiviral activity or the metabolism of the same class of nucleoside phosphoramidate prodrugs in NHBEs. To better understand the heterogeneity of lung tissue and guide lead compound selection during drug discovery, *in vitro* studies in various lung cells are warranted.

Using biochemical assays, we demonstrated efficient conversion of an intermediate metabolite MetX to the monophosphate by HINT1 enzyme. In addition, HINT1 was expressed in both lung S9 fraction and NHBE cells, indicating that HINT1 is likely involved in the activation pathway of RDV in the lung. While TAF MetX, as previously shown, was cleaved chemically under lysosomal conditions, RDV MetX was completely resistant to acid hydrolysis (5). Our biochemical studies confirmed that two reported HINT1 inhibitors, TrpGc and compound 7, effectively inhibit HINT1; however, neither of them significantly altered the intracellular formation of the downstream triphosphate metabolite in NHBE cells. Similarly, neither HINT1 inhibitor affected cellular activation of SOF (data not shown), a process that HINT1 has been shown to play a key role (7). These data indicate that the two HINT1 inhibitors may not function at the cellular level, possibly due to poor permeability or stability. However, we cannot rule out the possibility that some other unidentified enzymes may be contributing to the cleavage of the P-N bond. Nevertheless, we have demonstrated *in vitro* that HINT1 is, at least in part, involved in the activation pathway of RDV in the lung.

In this study, we also evaluated the expression of a series of enzymes potentially involved in the phosphorylation/dephosphorylation of RDV metabolites using bulk and single-cell RNA-seq public data sets. We did not experimentally profile these phosphotransferases based on the efficient formation of the TP metabolite observed in cell culture and preclinical animal studies, suggesting that the transformations of MP→DP and DP→TP are not rate limiting (31). Among nine isoforms of adenylate kinase, the enzyme that phosphorylates natural AMP to ADP, three of them (*AK1*, *AK2*, and *AK3*) were found to be highly expressed in many subsets of epithelial cells and some endothelial cells. Recently, Akinci et al. demonstrated that mitochondrial AK2 is able to phosphorylate the RDV monophosphate metabolite to the diphosphate metabolite using a recombinant enzyme. Knockout of the *AK2* gene in Huh7, a hepatoma cell line, resulted in a significant reduction in RDV anti-SARS-CoV2 activity, indicating that AK2 is involved in RDV activation (E. Akinci, M. Cha, L. Lin, G. Yeo, M. C. Hamilton, C. J. Donahue, H. C. Bermudez-Cabrera, L. C. Zanetti, M. Chen, S. A. Barkal, B. Khowpinitchai, N. Chu, M. Velimirovic, R. Jodhani, J. D. Fife, M. Sovrovic, P. A. Cole, R. A. Davey, C. A. Cassa, and R. I. Sherwood, personal communication). However, other nucleotide kinases may also contribute to RDV activation in lung cells. Phosphorylation of ADP or GDP to the corresponding triphosphate is facilitated by multiple enzymes involved in purine metabolism, including nucleoside diphosphate kinase (NDPK), phosphoglycerate kinase, creatine kinase, and pyruvate kinase (32). Among these enzymes, NDPK efficiently phosphorylates various diphosphates of antiviral nucleoside/nucleotide analogs. While we did not identify the particular enzyme that phosphorylates the RDV diphosphate metabolite, it is reasonable to assume that drug-drug interactions through inhibition of this step are unlikely because of the parallel pathways. In addition, our cellular and tissue RDV metabolism data showed a significantly lower RDV diphosphate metabolite level than the active triphosphate (33), indicating that the transformation of NDP to NTP is not rate limiting for RDV activation.

In conclusion, we demonstrated that CES1, CatA, and HINT1 are critical enzymes in the RDV activation pathway to its triphosphate species. These enzymes are not only highly expressed in the human lung but are also highly expressed in cell types that are involved in SARS-CoV-2 infection and replication. This information will enable a better understanding of potential drug-drug interactions, facilitate combination studies of RDV with other antiviral agents, and empower future antiviral drug discovery.

## MATERIALS AND METHODS

### Reagents.

RDV, its parent nucleoside GS-441524, metabolite intermediate MetX, 5′-triphosphorylated metabolite GS-443902, and positive-control compounds tenofovir alafenamide (TAF), GS-566650 (GS-6620 MetX), and GS-652829 (TAF MetX) were synthesized by Gilead Sciences, Inc. (Foster City, CA). Carboxylesterase 1b (CES1b), carboxylesterase 1c (CES1c), and carboxylesterase 2 (CES2) Corning Supersomes insect cell microsomal fractions containing individual baculovirus-expressed human CES enzymes were purchased from Corning Incorporated (Corning, NY). Postmitochondrial supernatant (S9) subcellular fractions were from BioIVT (Westbury, NJ). CES substrate 4-nitrophenyl acetate (4-NPA) was purchased from Sigma-Aldrich Inc. (St. Louis, MO). Cathepsin A (CatA), cathepsin L (CatL), and the fluorescent substrate, MCA-Arg-Pro-Pro-Gly Phe-Ser-Ala-Phe-Lys(DNP)-OH, were purchased from R&D Systems (Minneapolis, MN). CatL inhibitor E 64 was purchased from Tocris Bioscience (Minneapolis, MN). Telaprevir was obtained from Acme Bioscience (Palo Alto, CA). TrpGc and compound 7 were prepared by Wuxi AppTec (Tianjin, China). HINT1 was purchased from Abcam (Cambridge, MA). All other chemicals were purchased from Sigma-Aldrich (St. Louis, MO) or equivalent vendors. Internal standard/quench (IS/Q) used to stop reactions in metabolic incubations was 200 nM labetalol in 90% (vol/vol) acetonitrile, 10% (vol/vol) methanol, and 0.1% (vol/vol) formic acid or 200 nM labetalol in 90% (vol/vol) acetonitrile and 10% (vol/vol) methanol.

### Public gene expression profiling data sources.

### (i) Bulk mRNA expression data from normal human tissues.

The normalized gene expression data in transcripts per million (TPM) in nondiseased human tissue sites were downloaded from the Genotype-Tissue Expression (GTEx) (11) and the Human Protein Atlas (HPA) (12) databases, respectively. The GTEx data are based on the GTEx Analysis release v8, and the HPA data are based on the HPA version 20.0.

### (ii) Single-cell RNA-seq data from human lung and airways.

The metadata and gene expression matrix of unique molecular identifier (UMI) counts for two public single-cell RNA-seq data sets of normal human lung tissue (13) and human healthy airways (34) were downloaded using the BioTuring Browser software (v2.7.5) (BioTuring, Inc., San Diego, CA). The cell type information for individual cells in the metadata files were retrieved from the respective studies.

### Bioinformatics analysis of scRNA-seq data.

The scRNA-seq data were analyzed by R software (v3.5.2) (https://www.R-project.org/) with the Seurat package (v3.0.2) (14). In the first step, genes expressed in less than 5 cells were filtered out. Raw expression values for the cells were then normalized to 10,000 UMIs and log transformed by the NormalizeData function. Two thousand variable genes were identified by the FindVariableFeatures function with the vst method. A linear transformation of the data was performed by the scaleData function prior to dimensional reduction. The RunPCA function was used for linear dimensional reduction based on the scaled data. Finally, nonlinear dimensional reduction t-distributed stochastic neighbor embedding (tSNE) was performed on the top 20 principal components by the RunTSNE function. The R package ggplot2 (v3.3.0) was used for visualization.

### Cell cultures.

Primary human bronchial epithelial (NHBE) cells from several normal donors (donor identification 32027, 29744, 35382, 31689, and 29154) were purchased from Lonza (Walkersville, MD). Cells were maintained in bronchial epithelial growth medium (BEGM) containing all supplements provided in the BEGM bullet kit (Lonza). Experiments utilizing NHBE cells from all donors were conducted in cells between passages 3 and 5. The human alveolar epithelial cell line (A549) was maintained in a high-glucose Dulbecco’s modified Eagle’s medium (DMEM) supplemented with 10% fetal bovine serum, 1% penicillin/streptomycin, and 1% HEPES (Thermo Fisher Scientific). The A549-human angiotensin-converting enzyme 2 (hACE2) cells that stably express hACE2 were grown in the culture medium supplemented with 10 μg/ml blasticidin S (35). All cell lines used in these studies were maintained at 37°C with 5% CO_2_. All culture medium and antibiotics were purchased from Thermo Fisher Scientific (Waltham, MA). All cell lines were tested negative for mycoplasma.

### Quantification of CES1, CatA, and HINT1 mRNA expression in NHBE cells.

Cellular RNA of NHBE was extracted using Qiagen mini-RNA prep kits (Hilden, Germany). CES1, CatA, and HINT1 mRNA were quantified by TaqMan one-step quantitative reverse transcription-PCR (RT-qPCR) using QuantStudio 6 Flex from Applied Biosystems (Perkin Elmer, Foster City, CA). RNA was first transcribed into cDNA by reverse transcriptase from total RNA or mRNA. The cDNA was then used as the template for the qPCR. RT-qPCR mix was prepared following the vendor’s instructions, and each 20-μl reaction mixture contained 5 μl of 4× MasterMix, 1 μl of 20× tested primer probe, 1 μl of 20× control beta-actin primer, 5 μl of RNA, and 8 μl of distilled water. The following RT-PCR program was used: RT step at 50°C for 5 min, RT inactivation step at 95°C for 20 s for one cycle, denaturation step at 95°C for 3 s, and annealing/extending step to 60°C for 30 s for 40 cycles. The following primers and probes were ordered from Thermo Fisher Scientific: CES1, TaqMan gene expression assay ID Hs00275607_m1; CTSA, TaqMan gene expression assay ID Hs01563953_g1; HINT1, TaqMan gene expression assay ID Hs00602163_m1; and beta-actin endogenous control (VICTM/TAMRATM probe, primer limited, catalog no. 4310881E). Analysis of data is described under the “Data analysis” section.

### Measurement of CES1, CatA, and HINT1 protein expression in NHBE and human lung S9 and liver S9 fractions.

The levels of CES1, CatA, and HINT1 proteins were expressed as percentage of total protein (wt/wt). The cell lysate used to measure total protein concentration was prepared in the exact fashion as the samples prepared for quantification of the proteins of interests. Approximately 2 million NHBE cells were lysed in 0.1 ml of 1× cell lysis buffer (Cell Signaling, Danvers, MA) plus 1× protease inhibitors (Cell Signaling, Danvers, MA). Total protein concentration was determined using Pierce bovine serum albumin (BSA) assay protein kit (Thermo Fisher Scientific, Waltham, MA). Cellular levels of CES1, CatA, and HINT1 proteins were quantified using Western blot analysis. The amount of total protein loaded in each lane was adjusted to ensure the target protein signals fall into the linear range of the standard curves. Equal amounts of NHBE total protein from different donors were loaded into SDS-PAGE midi gel; 50 to 100 μg of total protein was used for CES1 detection, and 25 to 50 μg of total protein was used for CatA and HINT1 detection. In addition,0.2 to 10 μg of human liver S9 and lung S9 fractions and 2-fold serial-diluted recombinant proteins (1.56 to 100 μg) were loaded onto the same SDS-PAGE midi gel. After the gel was run for 1 to 2 h at 100 V with 1× morpholineethanesulfonic acid (MES) running buffer (Thermo Fisher Scientific, Waltham, MA), total proteins were transferred from the gel to the nitrocellulose membranes using iBlot 2 (Thermo Fisher Scientific, Waltham, MA). These nitrocellulose membranes were incubated with 10% milk-TBS-Tween buffer for 2 h at room temperature on an orbital shaker to prevent nonspecific binding, followed by incubation with specific primary antibody diluted in 5% milk-TBS-Tween buffer at 4°C overnight on an orbital shaker following the manufacturer’s instructions. The next day, membranes were washed 3 times, 5 min each, with 1× TBS-Tween on an orbital shaker, followed by incubation with specific conjugated secondary antibodies in the manufacturer’s recommended dilution of 5% milk-TBS-Tween buffer for 1 to 2 h at room temperature on an orbital shaker and three 5-min washes with TBS-Tween buffer. The chemiluminescence bands of the specific proteins on membranes were detected by charge-coupled-device (CCD) camera using ECL 2 developing reagents (Thermo Fisher Scientific, Waltham, MA). The bands of specific proteins were quantified by software using standard curves generated with recombinant proteins.

### Biochemical assays.

### (i) CES-catalyzed hydrolysis of RDV and positive-control compounds.

RDV or positive-control substrates (oseltamivir for CES1 or procaine for CES2) were incubated with individual Supersome preparations (final CES concentration, 1.5 mg/ml) in 0.1 M potassium phosphate buffer (pH 7.4) at 37°C. Substrates were added to a final concentration of 1 μM to initiate the reaction. The final incubation volume was 250 μl. Aliquots were removed after incubation for 0, 10, 30, 60, and 120 min. The reactions were stopped by the addition of the quench solution containing the LC-MS internal standard (IS) (labetalol). Following protein precipitation and centrifugation, 150 μl of supernatant was dried down and reconstituted with 250 μl water. All samples were analyzed by an LC-MS, and the analyte/internal standard peak area ratio (PAR) values were used for quantification. Disappearance of RDV or positive-control compounds was monitored over the incubation time period. Three experiments were conducted with two replicates in each experiment, and data are reported as averages from the three experiments.

### (ii) CatA-catalyzed hydrolysis of RDV and positive-control compounds.

CatA was activated following the manufacturer’s protocol. Briefly, 950 nM CatA precursor was incubated with 192 nM CatL in an activation buffer consisting of 25 mM MES (pH 6.0) and 5 mM dithiothreitol (DTT) for 30 min at 37°C. CatL was then inactivated by the addition of the cysteine protease inhibitor E 64 to a final concentration of 10 μM. CatA reactions were then initiated by the addition of RDV or TAF (positive-control substrate) at 1 μM in a 250-μl reaction volume consisting of 5 nM CatA, 25 mM MES (pH 6.5), 100 mM NaCl, 1 mM DTT, and 0.1% Nonidet P-40 (NP-40) at 37°C. At incubation times of 0, 1, 5, 10, 25, 45, 60, and 90 min, 25-μl aliquots of the reaction were added to 225 μl of ice-cold quench solution containing the LC-MS IS (labetalol) with 0.1% formic acid. Following protein precipitation and centrifugation, 150 μl of supernatant was dried down and reconstituted with 250 μl water. All samples were analyzed by LC-MS, and the peak area ratio of RDV or TAF to that of the IS was used for quantification. Three experiments were conducted with two replicates in each experiment, and data are reported as averages from the three experiments. The effects of inhibitors of various enzymes on the metabolism of RDV by CatA were studied, as described above, by including them prior to the addition of RDV substrate. Inhibitors tested were telaprevir (5 μM) and BNPP (50 μM). In these studies, the supernatants obtained following protein precipitation were directly diluted with an equal volume of water prior to LC-MS analysis.

### (iii) CES-catalyzed hydrolysis of 4-NPA and inhibition by inhibitors.

Threefold dilutions of compounds were prepared in 100% DMSO. Compounds (100 μM to 0.005 μM), CES isozymes, and substrate 4-NPA were mixed in a 100-μl reaction volume consisting of 100 mM potassium phosphate buffer (pH 7.4) and 1% DMSO. The final concentration of CES isozymes were 0.05 mg/ml CES1b with 6 mM 4-NPA (2 × *K_m_*), 0.05 mg/ml CES1c with 12 mM 4-NPA (2 × *K_m_*), and 0.1 mg/ml CES2 with 12 mM 4-NPA (2 × *K_m_*). The kinetics of product formation was measured by absorbance (wavelength, 405 nm) for 30 min at room temperature using a Molecular Devices M5 plate reader. The slopes of the progress curves were used to calculate reaction rates. Reaction rates were plotted as a function of inhibitor concentration, and the data were fit with a four-parameter logistic model using GraphPad Prism software (version 8; La Jolla, CA) to yield IC_50_ values.

### (iv) CatA-catalyzed hydrolysis of peptide substrates.

The reaction was studied under both pH 6.5 (to match CatA-catalyzed RDV reaction conditions with minimal acidic hydrolysis) and pH 5.5 (to mimic acidic lysosomal environment). CatA was initially activated following the manufacturer’s protocol. Briefly, 950 nM CatA was activated by incubation with 192 nM CatL in an activation buffer consisting of 25 mM MES (pH 6.0) and 5 mM dithiothreitol (DTT) for 30 min at 37°C. CatL was then inactivated by the addition of the cysteine protease inhibitor E 64 to a final concentration of 10 μM. Threefold dilutions of compounds were prepared in 100% DMSO. The final DMSO concentration was kept constant at 1% (vol/vol). Compounds (100 μM to 0.005 μM), 10 nM CatA, and 10 μM of the substrate, MCA-Arg-Pro-Pro-Gly Phe-Ser-Ala-Phe-Lys(DNP)-OH, were mixed in a 100-μl reaction volume containing either reaction mixture A (5 mM MES [pH 6.5], 100 mM NaCl, 1 mM DTT, and 0.1% NP-40) or reaction mixture B (25 mM MES [pH 5.5] and 5 mM DTT). The reactions were monitored over 30 min using a Tecan Spark plate reader (excitation, 320 nm; detection, 405 nm). The slopes of the progress curves were used to measure reaction rates. Reaction rates were plotted as a function of inhibitor concentration, and the data were fit with a four-parameter logistic fit using GraphPad Prism software to yield IC_50_ values.

### (v) HINT1-catalyzed hydrolysis of RDV MetX and positive control.

HINT1 reactions were initiated by the addition of substrate (1 μM) in a 250-μl reaction volume consisting of 250 nM HINT1, 20 mM HEPES (pH 7.2), 1 mM DTT, 1 mM MgCl_2_, 200 ng/ml BSA, and 0.1% NP-40 at 37°C. At incubation times of 0, 1, 5, 10, 25, 45, and 60 min, 25-μl aliquots of the reaction were added to 225 μl of ice-cold quench solution containing the LC-MS internal standard without 0.1% formic acid. Following protein precipitation and centrifugation, 150 μl of supernatants were dried down and reconstituted with 250 μl water. All samples were analyzed by LC-MS, and PAR values were used for quantification. Disappearance of the MetX substrate was monitored over the incubation time period. Three experiments were conducted with two replicates each experiment, and data are reported as averages from three experiments.

### (vi) Chemical hydrolysis of RDV MetX and positive control under acidic conditions mimicking lysosomal lumen.

To test the chemical stability of RDV MetX under acidic conditions (found within the lysosomal lumen) or neutral conditions (e.g., found in the cytosol of cells), 1 μM compound was incubated in a 250-μl reaction mixture consisting of 100 mM sodium acetate (pH 4.5) or 100 mM HEPES (pH 7.5). At incubation times of 0, 1, 10, 25, 45, and 60 min, 25-μl aliquots of the reaction were added to 225 μl of ice-cold quench solution containing the LC-MS internal standard without formic acid. For reactions carried out at pH 4.5, the pH of the reaction aliquots was raised to pH 9 to 10 by addition of 7.5 μl of 0.2 M NaOH prior to addition of the quenching solution. Following protein precipitation and centrifugation, 150 μl of supernatants were dried down and reconstituted with 250 μl water. All samples were analyzed by LC-MS, and PAR values were used for quantification. Disappearance of MetX was monitored over the incubation time period. Three experiments were conducted with two replicates for each experiment, and data are reported as averages from three experiments.

### Cellular metabolism in NHBE cells.

NHBE cells (5 × 10^5^) in 2 ml of BEGM media were seeded in each well of a 6-well plate and incubated overnight at 37°C and 5% CO_2_. The medium was aspirated and replaced with 2 ml of BEGM medium containing 1 μM RDV and the appropriate concentration of BNPP (2.5, 10, or 20 μM), telaprevir (2.5, 10, or 20 μM), HINT1 inhibitor compound 7 (0.5, 2.5, or 10 μM), or 0.2% DMSO, and the plates were incubated at 37°C and 5% CO_2_. Twenty-four hours post-compound addition, the cells were washed 3× with 1 ml of cold 1× Tris-HCl (pH 7.0)-buffered saline, and cells were harvested in 500 μl dry ice-cold extraction buffer (0.1% potassium hydroxide and 67 mM ethylenediamine tetra-acetic acid in 70% methanol, containing 0.5 μM chloro-ATP as internal standard) and transferred to a deep-well block on dry ice. The deep-well block was stored at −80°C until processing. At the time of harvest, two untreated wells for each donor were trypsinized, and the cell counts were determined. All compound combinations were tested in duplicate in cells from three donors.

### SARS-CoV-2 antiviral assay.

Antiviral activity of compounds against SARS-CoV-2 was evaluated as described by Xie et al. (36). Briefly, the human alveolar epithelial cell line (A549) overexpressing hACE2, A549-hACE2 cells, were plated into a white opaque 96-well plate (Corning) at 12,000 cells per well in phenol red-free medium containing 2% fetal bovine serum (FBS). On the next day, 3-fold serial dilutions of compounds were prepared in DMSO in a manner to normalize the DMSO concentration among all cell culture wells. The compounds were further diluted 100-fold in the phenol red-free culture medium containing 2% FBS. Cell culture fluids were removed and incubated with 50 μl of diluted compound solutions and 50 μl of SARS-CoV2-Nluc viruses (multiplicity of infection [MOI], 0.025). At 48 h postinfection, 50 μl Nano luciferase substrates (Promega) was added to each well. Luciferase signals were measured using a Synergy Neo2 microplate reader. The relative luciferase signals were calculated by normalizing the luciferase signals of the compound-treated groups to that of the DMSO-treated groups (set as 100%). The relative luciferase signal (*y* axis) versus the compound concentration (*x* axis) was plotted in software GraphPad Prism (version 8). The compound concentration for reducing 50% of luciferase signal (EC_50_) values was calculated using a nonlinear 4-parameter regression model. Three independent experiments were performed with technical duplicates.

### A549-hACE2 cytotoxicity analysis.

The cytotoxicity of compounds was determined in A549-hACE2 cells in the following manner. A549-hACE2 cells (12,000 cells per well in medium containing 2% FBS) were plated into a black opaque 96-well plate (Corning). The next day, 3-fold serial dilutions of compounds were prepared in DMSO in a manner to normalize the DMSO concentration among all cell culture wells. The compounds were further diluted 500-fold in the culture medium containing 2% FBS. Spent medium from overnight incubation was aspirated, 100 μl of diluted compound solutions were added to each well, and cultures were returned to 37°C with 5% CO_2_. After 48 h, 100 μl OneGlo substrate (Promega) was added to each well. Luciferase signals were measured using an Envision microplate reader. The relative luciferase signals were calculated by normalizing the luciferase signals of the compound-treated groups to that of the DMSO-treated groups (set as 100%). The relative luciferase signal (*y* axis) versus the compound concentration (*x* axis) was plotted in software GraphPad Prism 8 (version 8). The compound concentration for reducing 50% of luciferase signal as a measure of cell viability (CC_50_) values were calculated using a nonlinear 4-parameter regression model. Three independent experiments were performed with technical duplicates.

### Data analysis.

### (i) Calculation of IC_50_ values.

The IC_50_ values were defined as the concentration causing 50% decrease in product formation in the biochemical assays. Data were analyzed using GraphPad Prism 8.0. IC_50_ values were calculated by nonlinear regression analysis using the following sigmoidal dose-response (variable slope) equation (four-parameter logistic equation).

$$Y=100/\left[1+10^{\left({\text{logIC}}_{50}-X\right)}\times \text{HillSlope}\right]$$

where *X* is the log of the concentration of the test compound, and *Y* is the response. The IC_50_ values were calculated as an average of three or more independent experiments.

### (ii) Data analysis for mRNA determination.

The amount of CES1, CatA, and HINT1 mRNA in cells relative to a housekeeping gene, β-actin, was calculated using the formula Δ*C_T_* = *C_T_*_, gene of interest_ − *C_T_*_, β-actin_.

*C_T_*_, gene of interest_ and *C_T_*_, β-actin_ represent the cycle threshold values for the amplification of genes of interest (CES1, CatA, or HINT1) and β-actin, respectively, as determined by the computational analysis of amplification curves using the ABI Prism software. Calculation relative expression is calculated using the formula *R* = 2^−ΔΔ^*^CT^*.

### Statistical analysis.

The difference between different groups was analyzed using ordinary one-way analysis of variance (ANOVA; Sidak’s multiple-comparison test) (GraphPad Prism version 8.1). The differences between the dose-response of compound-treated groups and DMSO-treated group were compared using two-way ANOVA (GraphPad Prism version 8.1).

### Determination of RDV metabolism using LC-MS analysis.

### (i) Determination of RDV metabolism in biochemical assays.

In all studies apart from those with CES1 and CatA with enzyme inhibitors, samples (10-μl aliquots) were injected with a Thermo Scientific autosampler. The LC instrumentation consisted of a Thermo Scientific Dionex UltiMate 3000 RS pump, and the LC column was a Waters Acquity UPLC BEH C_18_ (1.7 μm particle size, 2.1 by 50 mm). Mobile phases A and B were water 99.9% (vol/vol) containing 0.1% (vol/vol) formic acid, and acetonitrile 99.9% (vol/vol) containing 0.1% (vol/vol) formic acid, respectively, pumped at 0.2 ml/minute. Elution was achieved by a series of linear gradients over 2.33 min followed by reequilibration for 2.57 min. The MS instrument was a Thermo Q Exactive Hybrid Quadrupole-Orbitrap operating in positive or negative ionization mode as appropriate. This was calibrated on a weekly basis, and data were processed with GMSU 8.4.66 software by Gubbs Inc. (Alpharetta, GA). Mass tolerance of 5 ppm was used.

### (ii) Determination of RDV activation in NHBE.

Each NHBE sample was treated with 500 μl of dry ice-cold extraction buffer (0.1% potassium hydroxide and 67 mM ethylenediamine tetra-acetic acid in 70% methanol, containing 0.5 μM chloro-ATP as internal standard). The above solution was vortexed for 5 min and then centrifuged at 20,000 × *g* for 10 min. The supernatant was transferred to a clean 96-deep-well plate and then dried on an evaporator. Once dry, samples were reconstituted with 80 μl of 1 mM ammonium phosphate buffer (pH 7) and transferred to a 96-short-well plate for analysis. An aliquot of 10 μl was injected into a Sciex QTrap 6500+ liquid chromatography-tandem mass spectrometry (LC-MS/MS) system run under the multiple reaction monitoring (MRM) operation mode.

Standard calibration curves for NHBE were constructed based on pmol of compound per sample. The standard curve was prepared by spiking an appropriate amount of GS-441524, its monophosphate and diphosphate metabolites, and GS-443902 solution, prepared in water into a blank NHBE matrix and then further diluting to complete the calibration line. Using the calibration curve, the amount of the metabolites was quantified in each sample, and the intracellular concentrations (pmol/million cells) were determined by dividing the value by the cell number in the sample.
