# Supplementary material for: Key Metabolic Enzymes Involved in Remdesivir Activation in Human Lung Cells
Source: Antimicrob Agents Chemother. 2021 Aug 17;65(9):e00602-21. doi: 10.1128/AAC.00602-21 (PMC8370248; doi:10.1128/AAC.00602-21)
Supplement: Supplemental file 1 — Fig. S1. Download AAC.00602-21-s0001.pdf, PDF file, 0.01 MB [file aac.00602-21-s0001.pdf]

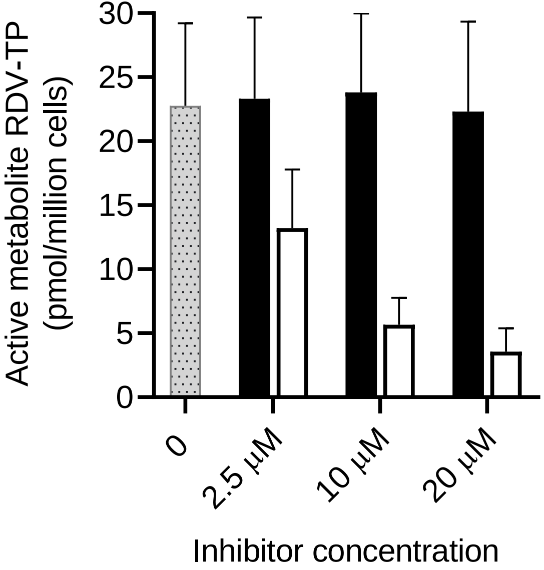

Figure S1. Replot of Figure 6A with different Y-axis units. Effect of CES1-inhibitor BNPP and CatA-inhibitor telaprevir on formation of active metabolite GS-443902 in RDV-treated NHBE. Cells from three different donors were incubated with 1 µM RDV and DMSO (grey dotted bar), BNPP (filled bar), telaprevir (open bar) and harvested at 24 hours post-compound addition. Triphosphate (TP) levels are shown as the average ± standard deviation pmol/million NHBE cells across the three donors. One-way ANOVA was used for statistical analysis.
